# Effect of pregnancy tests on demand for family planning: evidence from a randomized controlled trial in Uganda

**DOI:** 10.1186/s12978-021-01279-5

**Published:** 2021-11-15

**Authors:** Akito Kamei, Ryoko Sato, Rebecca Thornton

**Affiliations:** 1 University of Chicago, Chicago, USA; 2 Harvard University, 677 Huntington Ave, Boston, MA 02115 USA; 3 University of Illinois at Urbana-Champaign, Urbana, USA

**Keywords:** Pregnancy tests, Family planning, Unmet needs of contraception, Randomized controlled trial, Uganda

## Abstract

**Background:**

Unmet need for family planning and unintended pregnancies are high in developing countries. Home pregnancy tests help women determine their pregnancy status earlier and the confirmation of a negative pregnancy status can facilitate the adoption of family planning. This study provides the first experimental evidence of the effect of access to pregnancy tests on women’s demand for modern family planning.

**Methods:**

A randomized controlled trial was conducted among 810 women of reproductive age in northern Uganda. During a baseline survey, women were randomly allocated to either: (1) an offer to take a hCG urine pregnancy test during the survey (*on-the-spot* pregnancy test) (N = 170), (2) an offer of a home pregnancy test kit to be used at any time in the future (*future-use* pregnancy test) (N = 163), (3) offers of both *on-the-spot* and *future-use* pregnancy tests (N = 153), or (4) a control group (N = 324). Future-use pregnancy tests were offered either for free, or randomly assigned prices. Approximately 4 weeks after the baseline survey, a follow-up survey was conducted; modern contraception methods were made available at no charge at local community outreach centers.

**Results:**

When offered a free, *on-the-spot* pregnancy test, 62 percent of women accepted (N = 200). Almost all, 97 percent (N = 69), of women offered a free *future-use* pregnancy test strip, accepted it. Purchases of *future-use* pregnancy tests declined with price. The offer of either *on-the-spot*, *future-use* tests, or both, have no overall large or statistically significant effects on the take-up of modern family planning.

**Conclusion:**

Demand for pregnancy tests is high and access to pregnancy tests has the potential to facilitate the demand for family planning. At the same time, more research is needed to understand underlying beliefs about pregnancy status and risk that guide behaviors ultimately important for maternal and neonatal health.

*Trial registration* The study was pre-registered in July 2018 for AEA RCT registry (AEARCTR-0003187) and clinicaltrials.gov (NCT03975933). Registered 05 June 2019, https://clinicaltrials.gov/ct2/show/record/NCT03975933

## Plain Language Summary

Women in developing countries, especially in Africa, have high-unmet needs for family planning as well as high-unintended rates of pregnancy. At the same time, they may learn their pregnancy status later than women in higher-income countries due to irregular menstrual periods, malnutrition, or limited access to home pregnancy tests. Better awareness of pregnancy status can lead to the facilitation of family planning uptake. This paper experimentally evaluates the effect of the provision of home pregnancy tests on family planning take-up among Ugandan women. We find high demand for pregnancy tests among women when offered. At the same time, we find no impact of pregnancy tests on the take-up of free family planning at local community outreach centers. Demand for pregnancy tests is high and access to pregnancy tests has the potential to facilitate the demand for family planning. This study suggests that more research is needed to understand underlying beliefs about pregnancy status and risk that guide behaviors ultimately important for maternal and neonatal health.

## Introduction

Women in developing countries, especially in Africa, have high-unmet needs for family planning as well as high-unintended rates of pregnancy. Approximately 225 million women in developing countries have an unmet need for modern family planning [1]. Sedgh et al. [2] found that at least 25 percent of married women in 25 developing countries, 20 of which are in Africa, have unmet needs. In Africa, there are 89 unintended pregnancies for every 1000 women aged 15 to 44 [3]. Although unintended pregnancies have declined worldwide over time, the rate in developing countries is still much higher than in developed countries.

While earlier knowledge of pregnancy status can lead to the facilitation of family planning uptake [4, 5], women in developing countries may not learn that they are pregnant until later in their pregnancy due to irregular menstrual periods, malnutrition [6–10], or limited access to home pregnancy tests [11].

Home pregnancy tests have the potential to help women meet their needs of family planning by resolving the uncertainty of their pregnancy status. Confirmation of non-pregnancy status through pregnancy tests could allow women to access family planning—either due to their own increased motivation, or due to provider bias [12]. However, women in developing countries often face challenges in accessing home pregnancy tests, and there are often stock outs of tests at clinics [12]. This paper experimentally evaluates the effect of home pregnancy tests on family planning use.

Previous studies highlight the potential for pregnancy tests to facilitate increased adoption of hormonal contraceptives. Using a randomized experiment in Ghana and Zambia, Stanback et al. [5] studied the effects of supplying family planning clinics with pregnancy tests on service denial among non-menstruating women. In Zambia, the rate of denial decreased by over 70 percent; there was no effect in Ghana. In another randomized controlled trial that provided community health workers with pregnancy tests in Madagascar, Comfort et al. [12] found a 26 percent increase in women supplied with hormonal contraceptives. These existing studies suggest an important link between pregnancy tests and family planning adoption but do not determine the underlying pathways explaining their results. Does family planning use increase because health providers are more comfortable supplying women with hormonal contraception (eliminating provider bias), or do pregnancy tests affect women’s demand for family planning?

In this paper, we provide the first evidence of the demand for home pregnancy tests and measure the effect of access to pregnancy tests on the adoption of modern family planning, among sexually active women in northern Uganda who are not using modern contraceptive methods.

The study was conducted in Etam sub-county, Amolatar District, Lango sub-region, in Northern Uganda. Having experienced civil war since the early 1990s, Northern Uganda has been suffering from poor infrastructure, high poverty rates, and the lowest literacy rates in the country. Within the Lango region, Amolatar District has the lowest take-up of the family planning; the average take-up rate of the modern family-planning methods in 2016 was 24 percent in Amolatar district (Uganda Bureau of Statistics, 2018). Approximately 16 percent of women between ages 12–19 had given birth at least once in Amolatar District (Uganda Bureau of Statistics, 2017).

In Etam sub-county, Etam Health Center (HC3) is the main health provider of in-patient, diagnostic, and maternity services in the area. Free short-term family planning methods such as injection and condom are available at Etam HC3 [13], although in practice, there are challenges with maintaining continuously available commodities at public health centers and stock-outs are frequent. There is one private provider, Marie Stopes, who visits Etam HC3 every 3 months to provide free short- and long-term family planning.

Out of 209 health facilities studied in 2013 in the service availability readiness survey in Uganda, only 84 percent of the HC3s were able to provide family planning service. In terms of pregnancy testing kits, the same survey shows that only 53 percent had urine pregnancy test readily available [14]. At private clinics or local pharmacies, stock-outs are less frequent and pregnancy tests are available for 2000–3000 Ugandan Shillings (approximately 50–90 cents).

## Methods

The study was conducted between May and September 2019.

### Selection of participants

**Fig. 1:**
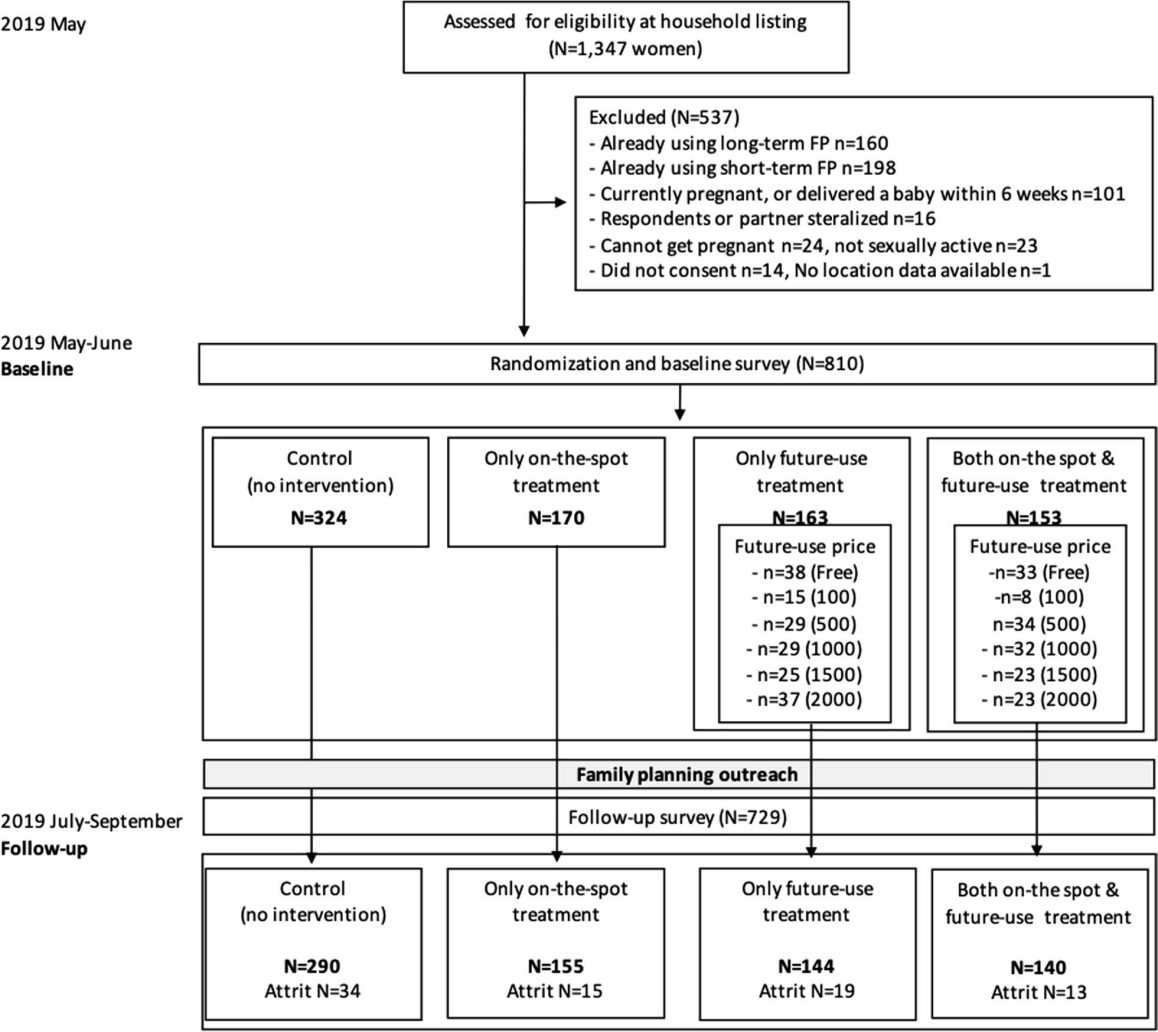
CONSORT figure: flowchart of sample selection and random treatment assignment

Our sample includes women living in 71 villages in the catchment area of Etam HC3. In each study village, we conducted a household listing to identify women eligible for the study. Figure 1 presents the CONSORT diagram.^1^

Women were eligible if they were between 18 and 35 years old, not currently pregnant, if they had not delivered a baby in the past 6 weeks, and had a current sexual partner. In households with multiple eligible women, one woman was randomly selected. We identified 1347 eligible women at the household listing.

Each woman was then further screened and excluded from the study if they did not consent for the baseline interview (N = 14), not sexually active (N = 23), reported being unable to get pregnant (N = 24), were sterilized (N = 16), were already using modern family planning (e.g., an implant, IUD, injectables, or pill; N = 358), or reported either being currently pregnant or having delivered a baby in the last 6 weeks (N = 101).^2^

Using these criteria, respondents in the study include women who face uncertainty about their pregnancy status and may have a demand for family planning. Our analysis sample includes a total of 810 women.

### Baseline survey

Enumerators conducted a face-to-face baseline survey to collect basic demographic and socio-economic information, past and current reproductive health behavior and family planning use. At the end of the baseline survey, women were given 2000 Shillings (approximately 50 cents) for their participation.

### Pregnancy test interventions

**Fig. 2:**
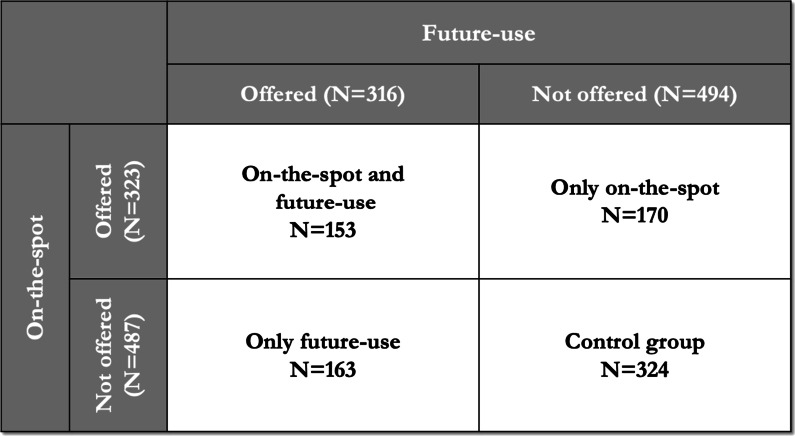
Research design: *On-the-spot* and *pregnancy testing for future use*. “On-the-spot" means women in this group were offered to take pregnancy testing service. Following the baseline survey, women in this treatment arm were offered the opportunity to take an on-the-spot pregnancy test. The pregnancy test consisted of a simple dip-strip urine-based pregnancy test together with an explanation of how to interpret the results with enumerators. “Future-use" means that they were offered pregnancy testing kit to be used in the future. Women in this treatment arm were offered a home pregnancy test kit to keep for their own use any time in the future. Enumerators explained how to use the pregnancy test kit and respondents were provided with both a single pregnancy test strip and informational a pamphlet explaining how to use the test and interpret the results

We conducted two interventions during the baseline survey, both randomized at the individual level (Fig. 2). Respondents were randomly assigned to each treatment arm through a random number generator within electronic tablets using the Open Data Kit program [15].

First, 323 women were randomly assigned to the *on-the-spot* pregnancy test treatment. Following the baseline survey, women in this treatment arm were offered the opportunity to take an on-the-spot pregnancy test. The pregnancy test consisted of a simple dip-strip urine-based pregnancy test together with an explanation of how to interpret the results with enumerators.

Second, stratifying by on-the-spot treatment assignment, 316 women were randomly assigned to the *future-use* pregnancy test treatment. Women in this treatment arm were offered a home pregnancy test kit to keep for their own use any time in the future. Enumerators explained how to use the pregnancy test kit and respondents were provided with both a single pregnancy test strip and informational pamphlet explaining how to use the test and interpret the results.

The cross-randomized design of two intervention creates four treatment arms, women who were offered (1) both *on-the-spot* and *future-use* pregnancy tests (N = 153); (2) only an *on-the-spot* pregnancy test (N = 170); (3) only a *future-use* pregnancy test (N = 163); (4) neither *on-the-spot* nor *future-use* pregnancy tests (N = 324).

While the *on-the-spot* test was offered for free, the *future-use* test kit was offered at a randomly assigned price ranging from free, up to 2000 Shillings. See Fig. 1 for the full distribution of prices across treatment arms.

### Family planning outreach centers

As part of the study, family planning services were provided by a local outreach organization, Reproductive Health Uganda (RHU). Respondents were informed of the locations and dates of the outreach services, available over 2 weeks at four different locations (3 days at each location). The provision of the family planning service at the outreach centers reduces the time cost to travel to receive family planning,^3^ At each outreach center, free family planning counseling and contraceptive methods (e.g., injectables, pills, condoms, IUDs, and implants) were available to anyone in the community, unconditional on their participation in the study.

### Outcome measure

Our outcome is whether a woman received any form of modern family planning (e.g., injectables, pills, IUD, or implant) at the local family planning community outreach center, 4 weeks after the baseline survey. We analyze this outcome among all 810 women in the study.

### Statistical analysis

We estimate the intention to treat effect of being offered an *on-the-spot* pregnancy test, a *future-use* pregnancy test, or both, on the adoption of modern family planning. We estimate the following logit regression model:

$$Y_{iv} = \alpha + \beta_{1} {\text{on - the - spot}}_{iv} + \beta_{2} {\text{future - use}}_{iv} + \beta_{3} {\text{both - tests}}_{iv} + X^{\prime}_{iv} \gamma + \varepsilon_{iv}$$

where $Y_{iv}$ is our outcome measures of family planning use for woman *i*, living in village *v*. *On-the-spot* is an indicator of being offered an on-the-spot pregnancy test, *future-use* is an indicator of being offered a future-use pregnancy test, and *both-tests* is an indicator of being offered both on-the-spot and a future-use test. The omitted category is the control group.

We present estimates that include a vector, *X*, of control variables, and estimates that omit these controls. Control variables include indicators of age in five-year increments, an indicator of living less than 5 km to the HC3, an indicator of being married and an indicator of primary school completion. Our coefficients of interest are $\beta_{1}$, $\beta_{2}$, and $\beta_{3}$, which we present as marginal effects. We present robust standard errors clustered at the level of a woman’s village (71 villages).

Comparing each treatment group (N =  ~ 320), to our control (N = 324), with 32 percent take-up of family planning at outreach in our control group, our study is powered to detect differences of 11 percentage points with power of 0.9 and significance level 0.05*.*

## Results

### Baseline characteristics and balance

Table 1 presents the characteristics of respondents at baseline among women in each of the treatment arms. The first two columns present the statistics for women in the control group. Among these 324 women, 27 percent were between 18 and 20 years old, 28 percent between 21 and 25 years old, 21 percent between 26 and 30 years old, and 23 percent between 31 and 35. The majority, 69 percent, had completed primary school. Women lived in households with one to four members (33 percent), five to seven members (43 percent), with fewer, 24 percent living in households more than eight member. More than half of women (54 percent) lived more than 5 km away from HC3.

**Table 1:** Baseline Characteristics and Balance Test in Etam sub-county in Northern Uganda, 2019

	Control		Only on-the-spot		Only future-use		Both	
	Freq (N)	Percent (%)	Freq (N)	Percent (%)	Freq (N)	Percent (%)	Freq (N)	Percent (%)
Age								
18–20	88	27	50	29	47	29	31	20
21–25	92	28	49	29	53	33	58	38
26–30	68	21	35	21	37	23	32	21
31–35	76	23	36	21	26	16	32	21
Complete primary school								
No	101	31	46	27	41	25	38	25
Yes	223	69	124	73	122	75	115	75
Household size								
1–4	107	33	64	38	63	39	59	39
5–7	139	43	70	41	64	39	60	39
8–24	78	24	36	21	36	22	34	22
Distance to health center 3								
Less than 5 km	148	46	69	41	74	45	66	43
5 km or more	176	54	101	59	89	55	87	57
Marital status								
Married with husband	246	76	140	82	135	83∗	118	77
Have a partner^a^	78	24	30	18	28	17	35	23
Number of sex in the last 4 weeks								
0	78	24	28	17	30	19∗	30	20
1–4	65	20	42	26	27	17	45	30
5–8	81	25	39	24	37	24	26	17
9–12	52	16	31	19	32	21	28	19
13–35	45	14	24	15	30	19	21	14
Desire for children								
Do not want any/more child	65	20	34	20	26	16	25	16
Want to have a/another child	215	66	108	64	116	71	107	70
Undecided/Don’t know	44	14	27	16	21	13	21	14
I am ready now	32	12	17	13	13	10	23	18
Within next year	15	6	7	5	13	10	6	5
1–2 years	64	25	28	21	41	30	30	23
3–4 years	59	23	38	28	33	24	32	25
5 years or more	79	31	39	29	33	24	31	24
After marriage	8	3	6	4	2	1	6	5
Family planning use								
Condom	41	13	17	10	15	9	14	9
Withdrawal	38	12	20	12	21	13	13	9
Rhythm method	56	17	41∗	24	29	18	33	22
Observations	324		170		163		153	

Observations at women level. The asterisk is associated to chi-square tests of categorical variables for each treatment arm compared to control arm at different significance level, ^*^p < 0.10, ^**^p < 0.05, ^***^p < 0.01 ^a^Have a partner but not living together

The majority, 76 percent were married, with the remaining having a partner but not living together. In the 4 weeks prior to the baseline, 24 percent of women had no sexual intercourse, with 55 percent having had sexual intercourse with their partner more than five times. On average, the majority, 66 percent report wanting more children, while only 12 percent are ready to have additional children at the time of survey. In terms of methods used to prevent pregnancy, 13 percent use condoms, with 29 percent using withdrawal or rhythm method.

Columns 3–8 present the statistics for the remaining three treatment arms; statistical significance from chi-square tests for each treatment arm compared to the control group are shown as asterisks with * indicating p < 0.10, ∗∗ p < 0.05, and ∗∗∗ p < 0.01. Baseline characteristics are statistically balanced across treatment arms. Out of the eleven indicators listed in Table 1, only three variables, marital status, number of times having sexual intercourses in the past 4 weeks, and use of rhythm method are statistically different from the control with a p-value less than 0.10.

### Pregnancy test take-up

**Fig. 3:**
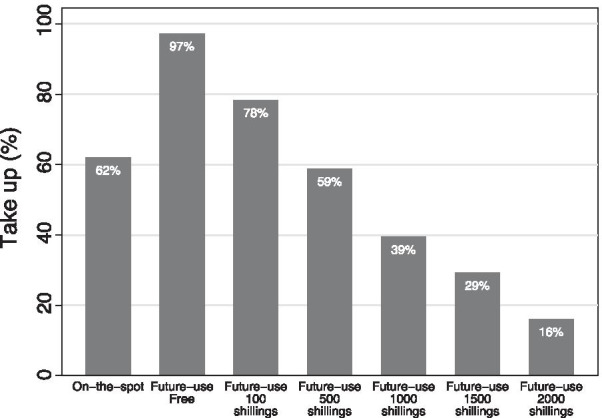
Average take-up of pregnancy testing kit. The figure shows the take-up of the intervention among women who were offered. Women in on-the-spot pregnancy test treatment were offered the opportunity to take a pregnancy test just after the conclusion of the survey. The pregnancy test consisted of a simple dip-strip urine-based pregnancy test together with an explanation of how to interpret the results with enumerators. Some women in future-use pregnancy test treatment were offered pregnancy test to keep for their own use any time in the future with the random price. The price offered was 100 (2.7 cents), 500 (14 cents), 1000 (27 cents), 1500 (41 cents), 2000 (51 cents) in Uganda Shillings (Number in parenthesis shows the price in US cents). Together with the single pregnancy test strip, we provide an informational pamphlet explaining how and when to use the test and interpret the results

Figure 3 presents the take-up of either *on-the-spot* or *future-use* pregnancy tests. Among women offered an *on-the-spot* pregnancy test, 62 percent accepted. Among women offered a *future-use* pregnancy test, the acceptance rates decreased with price ranging from 97 percent among women offered one for free to 16 percent offered a test at 2000 Shillings.

### Family planning take-up

Overall, 31 percent of women (N = 248), received family planning at the community outreach centers; among these 248, the majority received pills (64 percent) or injectables (24 percent).

### Effect of pregnancy tests on family planning take-up

Approximately 32 percent (N = 103) women from our control arm received family planning at our outreach (29 percent for only on-the-spot, 33 percent for only future-use, and 27 percent for both on-the-spot and future-use).

Table 2 presents the marginal intent to treat effects of the offer of on-the-spot, future-use, and both on-the-spot and future-use pregnancy tests on family planning outcomes. Column 1 present the results without controls, while Column 2 results with a vector of baseline controls. The results suggest that there are no large or statistically significant effects of being offered on-the-spot, future-use pregnancy tests, or both.

**Table 2:** ITT effect of pregnancy test provision on family planning uptake in Northern Uganda, 2019

	Received FP at outreach	
	(1)	(2)
Only on-the-spot	0.024 (0.043)	0.026 (0.044)
Only future-use	0.013 (0.044)	0.007 (0.043)
Both on-the-spot and future-use	−0.050 (0.044)	−0.051 (0.046)
Control	No	Yes
Control group mean	0.32	0.32
Observation	810	810

^*^p < 0.10, ^**^p < 0.05, ^***^p < 0.01. Standard error clustered at the village level (71 villages) is in parentheses. The table shows the marginal effect from the logit estimation. Control includes an indicator of baseline use of modern family planning, age of women in 5 years increment, completion of primary school, whether respondent resides with partner/husband, number of sex in the last 4 weeks, and desire for children

## Discussion

Uncertainty about pregnancy status faced by women of reproductive age might contribute to the low and delayed uptake of health services such as family planning and ANC, which could significantly affect reproductive and maternal and child health outcomes, especially in developing countries. This paper provides the evidence on how access to pregnancy tests affects family planning take-up. We conducted a randomized controlled trial to test the hypothesis that offering women home pregnancy tests affects the demand for family planning.

The study revealed that the demand for pregnancy tests is relatively high: among women offered an *on-the-spot* pregnancy test, 62 percent of women accepted it. The most common reason reported for not wanting a test was that women did not believe they had a chance of being pregnant. Women who experienced menstruation in the ten days before the baseline survey and intervention were 14 percentage points less likely to want a pregnancy test than those who experienced menstruation more than 10 days from the interview date (results available upon request).

Among the sample of women we study in Uganda, we found no large effects of offering pregnancy tests on modern family uptake at community outreach centers, 4 weeks after our intervention. Prior studies have shown a positive effect of giving health providers pregnancy tests on family planning adoption [5, 12]. For example, Comfort et al. [12] found that the provision of pregnancy tests among community health workers increased the uptake of modern contraceptives among women in Madagascar. Their study, however, did not identify the underlying mechanisms under which pregnancy tests increased the uptake of contraceptives. Access to pregnancy tests could increase family planning take-up either by removing provider bias or increasing the demand for the family planning among women, or both. Our study, on the other hand, focuses on the demand side because we directly offered pregnancy tests to women and observed women’s subsequent choices to adopt family planning at local outreach centers.

Our study has several limitations. First, our study presents intention to treat effects of pregnancy tests. Of those who were offered *on-the-spot* pregnancy tests, only 62 percent accepted. Similarly, not all women offered a *future-use* pregnancy test at a randomly assigned price acquired the test. Still, our point-estimates of the intention to treat estimates are small and we can rule out effects that are larger than 4.7 percentage points (*on-the-spot* only treatment) or 7.7 percentage points (*future-use* only treatment), at the 10 percent level. Second, due to logistical constraints, we were unable to offer family planning outreach services immediately after women were offered *on-the-spot* pregnancy testing. This may have reduced the value of the pregnancy test, especially if women then began to engage in risky sex. Exploratory analysis among a sub-sample of women who did not engage in risky sex after the baseline survey suggest positive demand for family planning, suggesting that this may, in part, be an important consideration. However, the gap in time between pregnancy testing and access to family planning at the community outreach centers more accurately represent the setting in northern Uganda, where there are delays in access to services. Future research that can combine pregnancy test and family planning provision may help to shed light on this further; the combination of family planning and pregnancy tests in the Madagascar study among community health workers may be one reason for the success of that that program [12]. Finally, the study was conducted in a specific area of Uganda, thus may not generalize to other settings.

## Conclusions

Demand for pregnancy tests is high and access to pregnancy tests has the potential to facilitate the demand for family planning. At the same time, more research is needed to understand underlying beliefs about pregnancy status and risk that guide behaviors ultimately important for maternal and neonatal health.

## Data Availability

The datasets used and/or analyzed during the current study are available from the corresponding author on reasonable request.

## Abbreviations

IUD: Intrauterine Device
RHU: Reproductive Health Uganda
FP: Family Planning
HC3: Health Center
ANC: Antenatal care
CHW: Community health workers

## Footnotes

1 This study follows CONSORT guideline. CONSORT 2010 checklist of information to include when reporting a randomized trial is available upon request.

2 We drop one additional woman from the study because her household location was not recorded.

3 The locations of the outreach centers were chosen so that the distance from respondents’ residential location to each outreach center did not correlate with treatment status. The average distance to the closest center from respondents’ home was 2.2 km and 90 percent of respondents lived within 3.7 km.

## References

[1] Singh S, Darroch JE, Ashford LS (2014). Adding it up: the costs and benefits of investing in sexual and reproductive health 2014.

[2] Sedgh G, Ashford LS, and Hussain R. "Unmet need for contraception in developing countries: examining women’s reasons for not using a method." New York: Guttmacher Institute 2 (2016): 2015–2016. http://www.guttmacher.org/report/unmet-need-for-contraception-in-developingcountries.

[3] Bearak J, Popinchalk A, Alkema L, Sedgh G (2018). Global, regional, and subregional trends in unintended pregnancy and its outcomes from 1990 to 2014: estimates from a Bayesian hierarchical model. Lancet Glob Health.

[4] Shelton JD, Angle MA, Jacobstein RA (1992). Medical barriers to access to family planning. Lancet (London, England).

[5] Stanback J, Vance G, Asare G, Kasonde P, Kafulubiti B, Chen M, Janowitz B (2013). Does free pregnancy testing reduce service denial in family planning clinics? A cluster-randomized experiment in Zambia and Ghana. Global Health Sci Pract.

[6] Gebre B, Biadgilign S, Taddese Z, Legesse T, Letebo M (2018). Determinants of malnutrition among pregnant and lactating women under humanitarian setting in Ethiopia. BMC Nutr.

[7] Harlow BL, Signorell LBO (2000). Factors associated with early menopause. Maturitas.

[8] Harlow SD, Matanoski GM (1991). The association between weight, physical activity, and stress and variation in the length of the menstrual cycle. Am J Epidemiol.

[9] Lindsay KL, Gibney ER, McAuliffe FM (2012). Maternal nutrition among women from Sub-Saharan Africa, with a focus on Nigeria, and potential implications for pregnancy outcomes among immigrant populations in developed countries. J Hum Nutr Diet.

[10] Rowland AS, Baird DD, Long S, Wegienka G, Harlow SD, Alavanja M, Sandler DP (2002). Influence of medical conditions and lifestyle factors on the menstrual cycle. Epidemiology.

[11] Morroni C, Moodley J (2006). The role of urine pregnancy testing in facilitating access to antenatal care and abortion services in South Africa: a cross-sectional study. BMC Pregnancy Childbirth.

[12] Comfort AB, Chankova S, Juras R, His CN, Peterson LA, Hathi P (2016). Providing free pregnancy test kits to community health workers increases distribution of contraceptives: results from an impact evaluation in Madagascar. Contraception.

[13] Ministry of Health, Uganda. 2014. Uganda Family Planning Costed Implementation Plan, 2015–2020. Kampala: Ministry of Health, Uganda.

[14] World Health Organization, Health statistics and information systems. Uganda service availability and readiness assessment 2013 Summary report: Key findings in figures. 2013; WHO: Geneva http://www.who.int/healthinfo/systems/sara_reports/en/.

[15] Hartung C, Lerer A, Anokwa Y, Tseng C, Brunette W, and Borriello G. Open data kit: tools to build information services for developing regions. In Proceedings of the 4th ACM/IEEE international conference on information and communication technologies and development; 2010. pp. 1–12.

